# Arts-based interventions for people living with dementia: Measuring ‘in the moment’ wellbeing with the Canterbury Wellbeing Scales

**DOI:** 10.12688/wellcomeopenres.16596.3

**Published:** 2021-09-09

**Authors:** Sarah Strohmaier, Karl M. Homans, Sabina Hulbert, Sebastian J. Crutch, Emilie V. Brotherhood, Emma Harding, Paul M. Camic

**Affiliations:** 1 Salomons Institute for Applied Psychology, Canterbury Christ Church University, Tunbridge Wells, Kent, UK; 2 School of Psychology and Life Sciences, Canterbury Christ Church University, Canterbury, Kent, UK; 3 Dementia Research Centre, Department of Neurodegeneration, UCL Queen Square Institute of Neurology, University College London, London, UK

**Keywords:** dementia, wellbeing, arts-based interventions, Canterbury Wellbeing Scales

## Abstract

**Background: **There is growing acknowledgement for the need to move beyond exclusive biomedical understandings of dementia and also focus on how to improve the lives and wellbeing of people living with dementia. A mounting body of research advocates for the benefits of arts-based interventions for this population. The purpose of this study was to explore the links between multiple components of arts-based interventions and subjective wellbeing in order to help assess if these activities might contribute to meaningful community-based dementia care initiatives.

**Methods: **Using previously collected data across different intervention sites, a within- and between- participants design was used that assessed wellbeing through the Canterbury Wellbeing Scales (CWS) in people with mild-to-moderate dementias (N = 201) who participated in various community arts-based interventions (ABI). Data were analysed using non-parametric statistical analyses and bootstrapped moderation models.

**Results:** Increases in subjective wellbeing were associated with all forms of ABI. Co-creative sessions significantly strengthened the relationship between number of sessions attended and overall wellbeing as well as optimism. No significant moderating effect was observed between number of sessions attended and carer presence.

**Conclusions: **In the largest study of its kind to date to assess wellbeing using arts activities in a community-based dementia sample, findings support the use and acceptability of the CWS as a measurement tool for people with early-to-middle stages of dementia and suggest that the CWS can reliably measure wellbeing in this population. In addition, the positive effect of arts-based interactions on specific aspects of wellbeing were found, which provide a better understanding of the conditions under which these effects can be prolonged and sustained. Further research is needed to better understand the environmental, social, and psychological mechanisms through which these improvements operate.

## Introduction

### Dementia

Dementia is an umbrella term that refers to a collection of syndromes rather than a singular disease affecting memory, cognition and behaviour, and has a substantial impact on an individual’s daily functioning ( Alzheimer’s Association, 2019). With significant personal, societal and economic consequences, dementia is one of the major causes of disability among older people and those with younger onset, and is an increasing global health concern ( WHO, 2017). In the absence of effective disease-modifying pharmacological treatments for dementia there is a growing acknowledgement of the need to move beyond biological interventions with a greater focus on the social context of people with dementia and carers to promote wellbeing ( NICE, 2019).

### Wellbeing in dementia

 The concept of personhood in dementia ( Kitwood, 1997) suggests that people can achieve a state of relative wellbeing if an individual is able to maintain their personhood through person-centred care ( Kitwood & Bredlin, 1992), whereby the individual’s subjective experience is acknowledged as *their reality* ( Brooker, 2003). In order to maintain personhood and achieve a sense of subjective wellbeing ^1^, Kitwood proposed that five areas of need should be addressed: comfort, attachment, inclusion, occupation and identity. Wellbeing is one of several areas of study considered within the conceptual framework of positive psychology, which is an approach that examines human strengths, assets, and capabilities ( Seligman, 2011) as opposed to medical and dominant psychological models that attend to problems, deficits, and treatments.

There has been a long history of research and measurement of wellbeing ( Diener *et al.*, 1999) yet many of the attempts to develop further understanding have led to numerous descriptions of dimensions rather than providing a definition. Two main schools of thought exist regarding what constitutes wellbeing: hedonic and eudaimonic. The hedonic assumes wellbeing can be maximised through pleasurable experiences and positive affect ( Diener, 1984) whereas the eudaimonic expands on this, proposing that wellbeing is not only about pleasure but involves Maslow’s (1968) need for self-actualisation ( Ryff, 1989). Despite the apparent differences, there is some consensus emerging that wellbeing is a multi-dimensional construct that encompasses both approaches ( Diener, 2009). Within dementia research a recent review documented the development of outcome measures based on positive psychological theories, and whilst promising advances have been made, there remains the need for further research in this area ( Stoner *et al.*, 2019).

In their attempt to define wellbeing, Dodge *et al.* (2012) consider wellbeing as multi-dimensional and posit that wellbeing is a fluctuating state or “see-saw” (p. 230) between an individual’s resources (psychological, social and physical) and challenges (psychological, social and physical). Due to the degree of changing difficulties associated with dementia it has been suggested that Dodge *et al.*’s fluctuating states theory may be particularly salient for dementia ( Camic *et al.*, 2019). Fluctuating states theory, as the name implies, provides a theoretical base to explore the changeable nature of wellbeing in dementia. It is one way to understand and appreciate “in the moment” experiences of wellbeing; experiences that may come about from engaging in, for example, a range of creative and interactive activities (e.g. the arts, sports, gardening, playing with pets) that shift perception ( Tipper, 2013) depending on the interests of the person living with dementia. According to Keady *et al.* (2020, p. 4-5), “in such instances, a moment can become a basic unit for creative expression and provision, isolated from external influences and interferences, and sustained through interactional processes of meaningful exchange.”

### Arts-based interventions

Despite the increased acknowledgement of a need to move away from a purely medicalised approach to dementia care (e.g. Kitwood, 1997; Zeilig *et al.,* 2014), there remains a reliance and assumption that care is often confined to the clinical settings of memory and mental health clinics ( Camic *et al.*, 2019). However, a World Health Organisation (WHO) report (2019) recommended that to improve the wellbeing of carers and those with dementia, people should be encouraged to participate in arts activities of their choice and have the opportunity to be creative and maintain social relationships. Recent reviews into the use of arts-based interventions (ABI) suggest that they can: aid communication, help maintain residual abilities, promote new learning, enhance cognitive functioning, increase confidence and self-esteem, improve social participation, improve psychological health, reduce behavioural symptoms and improve wellbeing ( Ander *et al.*, 2013; Beard, 2021; Clare & Camic, 2020; Young *et al.*, 2016).

Kaufmann & Engel (2016) echo Beard’s (2021) earlier conclusions and suggest that those with dementia are important informants of their wellbeing. Subsequent research has started to address this gap in the literature. A quasi-experimental study investigating museum object handling and wellbeing across early-to-middle stages of different dementias found significant improvement in wellbeing regardless of diagnosis or severity, with those in early stages demonstrating greater improvements ( Camic *et al.,* 2019). Similarly, in an earlier crossover study comparing the impact on wellbeing between museum object handling, art viewing and a non-art social activity significant increases in wellbeing following both arts interventions were found but not for a social-only activity ( Johnson *et al.*, 2017).

 Other ABI involving art viewing have reported promising results. In the first of a kind randomised controlled trial (RCT), a comparison between a guided art tour followed by art making and independent museum visits, significant increases in wellbeing were observed following the guided tour group ( Schall *et al.*, 2018). A further quasi-experimental study exploring the impact of object handling on wellbeing found increases in positive affect, wellness and happiness and a decrease in negative affect across different groups of older people ( Thomson & Chatterjee, 2016).

Similar increases in wellbeing for people with dementia have been reported for other forms of ABI where carers have also been present. For example, Bourne *et al.* (2019) reported increases in wellbeing for both dyad members following a singing and an art viewing session, with the singing group also demonstrating decreases in self-reported stress. Similarly, in a study using music and dance, increases in wellbeing were also found for both dyad members ( Zeilig *et al.*, 2019).

 Whilst the reported findings from the aforementioned studies suggest that ABI have a positive effect on wellbeing, there are limitations. Most studies included small-to-moderate sample sizes and there was little consistency regarding the definition and measurement of wellbeing. It therefore cannot be implied that the same conceptual phenomenon is being measured. The extant literature supports the suggestion that ABI can be used as viable social prescribing initiatives ( Chatterjee *et al.*, 2018) with positive impact to the wellbeing of people with dementia ( Young *et al.*, 2016). Furthermore, it has been postulated that the focus of participative art projects not only promotes wellbeing but also has positive implications for health, cognitive processes and communication ( Zeilig *et al.*, 2019). It is not clear, however, to what extent various components of ABI are associated with wellbeing. Furthermore, there is limited research that explores if there is a relationship between single and multiple sessions of ABI. The current study aimed to better understand both of these areas by exploring the following hypotheses.

### Hypotheses

H1) Wellbeing scores will increase following all forms of ABI; (H2) the number of ABI sessions attended is positively associated with subjective wellbeing; (H3) the relationship between ABI and wellbeing scores at post-session (when controlling for scores at baseline) will be stronger for ABI involving co-creativity; (H4) the relationship between number of sessions attended and wellbeing scores at post-session (when controlling for scores at baseline) will be moderated by whether participants were accompanied by a carer, with the relationship between ABI and wellbeing found to be stronger for those accompanied by a carer; (H5) there is a significant relationship between the type of dementia and pre/post session changes in subjective wellbeing.

## Methods

Using previously collected data across different intervention sites, a within- and between- participants design was used.

### Dataset and procedure

The study used previously collected data sets exploring the associations between ABI and subjective “in the moment” wellbeing ( MacPherson *et al.*, 2009). Data sets were held in password protected and encrypted university files by EB, PC and SS. Interventions were selected from community-based settings involving a range of arts activities for people with dementia that are often available in many locations across the UK and in other countries. Researchers EB, PC, SC, EH and SS were in direct contact with research sites to oversee the protocol and facilitate data collection. Participants included in the data sets were drawn from seven different community organisations from 2015–2019 ( Table 1). A range of arts-based interventions were used (singing, dancing, music making, museum object handling, art viewing and art making) ( Box 1), led by arts and heritage facilitators experienced in working with people with dementia. Interventions ranged from 60 to 120 minutes with those over 60 minutes offering a comfort break. The Canterbury Wellbeing Scales (CWS) were administered across all intervention sites in an identical manner. Immediately before and immediately after each intervention, the lead researcher or facilitator at each site read the directions for each subscale. Participants were provided a pencil or pen and shown the lines to mark their responses. If a participant was unable to physically make a mark, they were asked to place their finger on the line where they would have marked, and one of the facilitators or researchers made the mark and confirmed it was in the correct place. Ethical approval was obtained by a Canterbury Christ Church University ethics panel for all the interventions (approval 075\Ethics\2015-19). All participants provided written informed consent.

**Table 1.** Details of participants and interventions from each of the study sites.

Location (number of sessions per intervention)	Age range: PwD (mean)	Gender (carers)	Participants	Diagnosis	Intervention
Specialist science museum (3)	61–86 (69)	Female = 1 Male = 3	PwD = 4	AD = 2 FTD-fv = 1 FTD-bv = 1	Object handling
Multi-use creative space (4)	Not recorded	Female = 4(1) Male = 1(2)	PwD = 5 Carer = 3 Artist = 5	AD = 1 PCA = 2 NS = 2	Singing
Art gallery and performance space (studio) (7)	73–85 (78.2)	Female = 6(7) Male = 3(3)	PwD = 9 Carer = 10	AD = 4 Vascular = 2 FTD = 1 Mixed = 2	Art viewing and singing
Regional museum and art gallery (2)	58–85 (74.0)	Female = 11 (26) Male = 25(4)	PwD = 36 Carer = 30	AD = 16 Vascular = 4 FTD = 5 Mixed = 8 YOAD = 3	Object handling and art viewing
Local museum (1)	54–89 (74.8)	Female = 27 Male = 53	PwD = 80	AD = 37 Vascular = 24 FTD = 4 Mixed = 13 HIV = 2	Object handling
Concert hall rehearsal room (1)	62–91 (76.5)	Female = 4(3) Male = 6 (10)	PwD = 10 Carer = 7	AD = 6 FTD = 2 Mixed = 1 LB = 1	Singing
Large art gallery (1)	45–91 (70.1)	Female = 2 (4) Male = 4(3)	PwD = 6 Carer = 7	AD = 4 FTD = 1 Mixed = 1	Art viewing
Local arts venues (M = 2.6, range = 1–7)	56–95 (76.3)	Female = 16(22) Male = 17(8)	PwD = 33 Carer = 30	AD = 16 Vascular = 5 Mixed = 4 NS = 7 YOAD = 1	Music/dance Singing Music making/singing
Regional choir (12)	31–87 (70.9)	Female = 8 Male = 10	PwD = 18	AD = 6 Vascular = 1 FTD = 2 Mixed = 6 PDD = 1 SVD = 1 YOAD = 1	Singing

Key: PwD – people with dementia, AD – Alzheimer’s disease, PCA – posterior cortical atrophy, FTD – frontotemporal dementia, FTDbv - frontotemporal dementia behavioural variant, FTDfv – frontotemporal dementia familial variant, SVD – Small vessel disease, PDD – Parkinson’s disease dementia, HIV – human immunodeficiency viruses, LB – Lewy bodies, YOAD – young onset Alzheimer’s disease, NS – diagnosis not stated by participant.

**Box 1. Intervention details**

**Table:** 

Setting	Intervention description
Specialist science museum	The intervention consisted of three object handling sessions (60 minutes each), occurring over three weeks on the same day and time each week. The CWS ^1^ was administered immediately before and after each session. Refreshments were provided pre and post object handling. Object handling sessions took place seated around a rectangular table in a well-lit private room in the museum. Sessions were led by two facilitators who had received dementia awareness training. Three researchers observed all sessions unobtrusively at a distance from the back of the room. Different objects were used for each session and were picked to be novel and diverse in their cultural, historical and sensory qualities. Some were from the museum’s handling collection and others were contributed by one of the researchers. Facilitators passed one object at a time and initially generated discussion through asking a range of questions to encourage participation and exploration before sharing information about each object. These questions included sensory, tactile, visual and historic (e.g. What does this object this feel like? What smell does this remind you of? What is the function of this object? Do you like the way it looks? Is it old or new? Real or a reproduction?). At the end of the final session, the group curated a display of all the objects used in the study that was available for public viewing for one month. Objects included, for example, an 18 ^th^ c French glass floor protector (feet) for furniture, a 21 ^st^ c obsidian mirror, an unusual looking and not readably identifiable late 20 ^th^ c American artist-made ceramic salt shaker, an agate slice, a mid-20 ^th^ c Sumatran woven basket, spices such as clove, black pepper and turmeric, a 19 ^th^ c British iron metal key to open water and gas manhole covers, and Vietnamese fishermen’s glass floats. A handout was provided after each session consisting of photos and information on the objects explored and the time and date of the next session.
Multi-use creative space	The intervention included four one-hour co-creative arts sessions with a group of five people with a dementia, three partners, five artists (musicians and dancers), and two researchers. Attendance to sessions varied due to health difficulties but there were always at least three people with a dementia and two partners. Sessions took place weekly in a multi-use creative space with refreshments offered as participants entered. A large circle of chairs was set up around the outside of the room and a table with instruments was set up on one end of the room. All participants of the session were invited to engage in unstructured improvisatory music and dance to facilitate non-verbal communication without there being any preconceptions or expectations. The instruments selected were easily accessible since they did not require high-level technical skills or previous experience playing them. Instruments included hand chimes, tambourines, drums, Baoding balls and Kalimbas. In addition to using the instruments as they were intended, participants also used instruments in non-traditional ways to produce a sound. The session involved iterative dialogue characterised by reflective discussion after every session. Multiple sources of data were collected including dialogic interviews, video data in addition to CWS ^1^ scores, which were administered before and after every session by the research team.
Art gallery and performance space (studio)	The intervention was comprised of seven 120-minute practice sessions, one rehearsal session, and a public recital. Data were only collected at the seven weekly practice sessions because it was felt this might interfere with the rehearsal session, and would be impractical on the night of the public concert when family and friends were present. The practice sessions took place at various galleries within an historic, urban art gallery and within a studio performance space at the gallery, the latter being a room where lyrics were written, songs practiced and refreshments served. Participants were seated in a semi-circle, along with two accompanying musicians and two volunteers, in the studio space, and also seated or standing (by choice) when in one of the galleries. Several different but integrated components, involving all participants, were part of each session: as participants entered the studio space and became settled, the CWS ^1^ was administered. Refreshments and socialising then occurred at the beginning of each session for about 10 minutes, followed by a short walk (five minutes) to one of the galleries to view and discuss one painting each week for about 30 minutes (led by an experienced art historian and educator). Discussions included making observations and identifying themes from the paintings whilst viewing them in one of the galleries. After returning to the studio space and a taking comfort break (10 minutes), words/phrases were identified that best captured the observations (15–20 minutes). This first occurred in small groups and then shared across the larger group. A professional lyricist then helped to co-author with participants, lyrics based on the themes and observations, and composed music to match the lyrics each session. New songs were created and practiced each week (40–45 minutes). At the end of each practice session the CWS was again administered.
Regional museum and art gallery	Sessions were about 115 minutes and were divided into three parts: the object handling session (45 minutes) occurred in an activities room in the museum where 6-8 objects were presented; a 25-minute socialising refreshment break in an adjacent room; and the third part of the intervention was an art viewing session (45 minutes), occurring in one of the museum’s art galleries. Sessions were counterbalanced where 50 percent of participants began with the object handling session and 50 percent with art viewing. The CWS ^1^ was administered four times: before and after object handing and before and after art viewing. Sessions were led by an experienced museum educator who had received dementia awareness training, and assisted by an undergraduate psychology student intern and a volunteer. A researcher was also present at all sessions. The average size of groups were six people (three people with dementia and three carers) ranging from four to eight people. Objects were presented one at a time and people had the opportunity to hold, examine, and talk about them as a group as they were passed round. Questions about impressions of the objects included sensory descriptions, preferences, and reflections; associations and anecdotes were encouraged (e.g. What do you think it is made of? How does the object make you feel? How old do you think it is? Does it remind you of anything?). A wide range of objects were used (e.g. Victorian carbolic soap, ancient Egyptian scarab stone, Iron Age axe head, geode, 19th-century African headdress rest, fossilized shark’s tooth, 18th-century tinderbox). Art viewing comprised viewing selected paintings in the gallery and the facilitator’s use of open questions to discuss colour, texture, aesthetic preferences, and speculation on the artist’s intent. Paintings from different time periods (18 ^th^- 21 ^st^ c) were selected which had different content and styles, and a potential for visual discovery rather than obvious historical knowledge. Johnson *et al.* (2017).
Local museum	Sessions were 55–75 minutes, occurred in a public room of the museum where 5-6 museum objects from the collection, chosen by a museum educator who had received dementia awareness training, were presented to groups of 5-8 people with dementia. In addition to the facilitator, two staff from the local Alzheimer’s Society office were present along with one researcher. The CWS ^1^ was administered by a researcher and immediately before and after each session. The procedure for each session was similar: After facilitator and participant introductions, each object was first passed around to all participants so they could tactically experience it and have a closer look. Rather than lecturing about the objects, the sessions were question-led by the facilitator to participants. These non-memory-related questions helped to focus discussion and involve everyone. Sample questions for each object (with some variation) included: What do you think this object is? Any guesses about its age? What is it made of? Where is it from in the world? Would you give it as a gift if you owned it? If it were in your home, where would you keep it? What do you think about its texture, colour shape? Does it feel light or heavy to you? Participants were also encouraged to ask questions and make comments. Objects included: a tiger’s skull, fossilised seaweed, Victorian candle snuffer, preserved cotton bud, Stone Age New Zealand hand axe, Egyptian mummy wrapping sample, 19th-century biscuit tin, Islamic porcelain, Roman mosaic floor and Tunbridge Ware). Camic *et al.* (2019)
Concert hall rehearsal room	The data collection session was part of a newly formed, ongoing singing group in a large, urban setting for people with dementia and carers, and occurred during the sixth group session. Sessions were 60 minutes and led by an experienced choral conductor and accompanied by a pianist. The conductor was also an experienced facilitator who had previously worked with older adult populations. Immediately after administration of the CWS1, the session began with a welcome song, initiated by the conductor and pianist as an indicator for group members to move to the two rows of seats as the session was starting, they also joined in with singing as they moved. Following this, participants engaged in physical (e.g. stretching) and vocal warm up exercises and sang three songs in both sitting and standing positions, for those who were able to do so. The songs had previously been either partly or fully practiced in previous sessions. The choral group focused on vocal production and technique as well as exploring repertoire from sea shanties to opera. At the end of the session and after the CWS was administered, refreshments were served.
Large art gallery	Facilitated by an experienced gallery educator, the sessions took place in a large, urban art gallery and consisted of a tour of the gallery (60 minutes) to familiarise participants (people with dementia and carers) with the setting in the first session, when no data were collected, followed by a second visit to the gallery (75 minutes) two weeks later when CWS ^1^ data were collected. The data collection session took place in an airy, large art-filled room, and consisted of a PowerPoint presentation of 14 paintings from the gallery’s collection (75 minutes) where the facilitator described the paintings’ history and engaged the group by asking questions. Paintings from the 17th and 18th centuries were shown in pairs and people were asked to interpret and identify links between paintings. Visualisation techniques were also used (e.g. imagine you are in this scene, what can you hear/see/smell? What time of day do you think it is?). In addition to the facilitator, two researchers and two volunteers from the museum were also present. Refreshments were served after data collection was completed.
Local arts venues	The sessions all took place in six locations in the east of England, in dedicated rooms in community venues. Refreshments were available as people arrived and during a break in the session or at the end, depending on the activity. Six, twice monthly, two-hour sessions across three months were run for different activities. It was designed as an ongoing programme to allow people to attended regularly. New participants were welcomed at any point of the programme. Each workshop offered arts activities by experienced artist-facilitators who were assisted by a volunteer. All the workshops were music and/or movement themed and included, for example, African drumming, digital music making, Egyptian dance, and community dance (community dance is not confined to any specific type of dance and is concerned with engaging people creatively and safely in a dance style, or exploring dance ideas and forms of their own). An external evaluator attended all groups and collected CWS ^1^ data. Groups included older people with and without dementia, sometimes accompanied by a care but other times attending on their own. Only data from participants with dementia are reported here.
Regional choir	Sessions were a variety of group choral activities lasting between 50–105 minutes (not including breaks) and took place at purposely-built performing arts venue on a university campus, with the exception of one session which took place in a recording studio. Each choral session broadly followed a similar procedure: welcome and refreshments followed by CWS1 administration, choral activities (e.g. warm up exercises, singing familiar and unfamiliar songs, singing in smaller breakout groups and soloist performances) and post-choral refreshments after second CWS administration. Data were collected fortnightly, at six of the 12 sessions. Examples of songs sung included “In My Life” The Beatles “Stand By Me” Ben E King, “Don’t Be So Hard On Yourself” Jess Glynne (unfamiliar song). An experienced choral conductor led the sessions, assisted by the Alzheimer’s Society Singing for the Brain local lead. Two researchers were present at all sessions.

^1 ^Canterbury Wellbeing Scales.

### Participants

Participants ( *N* = 363) consisted of people with dementia and their carers (where applicable). All were living in the community with family members or on their own. People were approached by researchers or community partners and given an information sheet with details about the study. After reading the information sheet and if they indicated interest in participating, a meeting was arranged to further explain the intervention, the research project, and answer questions. At this second meeting, if they agreed to participate, informed written consent was taken. For the purpose of the current study, only results from the dementia group were extracted for a total of 223 participants. Once incomplete data was removed, a dataset of 201 participants was identified ranging in age from 31 – 95 years (M = 72.47), with five participants not providing age data. Although all participants acknowledged receiving a diagnosis of dementia from an NHS physician, nine declined to specify the type of dementia. Level of dementia impairment was classified using the global scores of the Clinical Dementia Rating Scale (CDR), which assigns a dementia severity range from 0 to 3 (with .5 – 1.0 representing early to middle stages ( Morris, 1997)) based on an individual’s cognitive and functional performance of memory, orientation, judgment and problem solving, community affairs, home and hobbies, and personal care ( O’Bryant *et al.*, 2008) or approximated from Mini Mental State Examination (MMSE) scores, which has been shown to be a valid surrogate measure of CDR ( Perneczky *et al.*, 2006).

Inclusion criteria: participants i) had been formally diagnosed with any type of dementia within the mild-to-moderate range of impairment, ii) were able to understand the nature of the research project and able to give written consent, iii) were able to participate in the designated arts-based intervention and iv) could understand and respond in English. Exclusion criteria: i) being unable to participate in a group environment or ii) having significant additional health problems (e.g. medical condition that was life threatening or physically disabling; a severe, disabling mental health problem such as psychosis, major depression). Demographics are detailed in Table 2.

**Table 2.** Demographic information for participants in dataset included in the current analysis.

Characteristic	Participants	Range	Mean
Gender Female Male	81 120		
Age		31–95	74.06
Type of dementia AD FTD Vascular Mixed YOAD HIV PCA PDD SVD LB Declined to identify	92 17 36 35 5 2 2 1 1 1 9		
Session type Object handling Music/dance Art viewing & singing Singing Art viewing lecture Object handling & art viewing Music making/singing	84 19 9 40 6 36 7		
Sessions co-created Yes No	75 126		
Carer present Yes No	86 115		

Key: PwD – People with dementia, AD – Alzheimer’s Disease, PCA – Posterior cortical atrophy, FTD – Frontotemporal Dementia, SVD – Small vessel disease, PDD – Parkinson’s Disease dementia, HIV – Human immunodeficiency viruses, LB – Lewy Bodies, YOAD – Young onset Alzheimer’s Disease.

### Measures

Participants completed the CWS ( Camic, 2020; Johnson *et al.*, 2017) before and after an arts-based intervention. Both researchers and community facilitators administered the CWS after a training session with one of the authors. The CWS is a visual analogue scale (VAS) consisting of five sub-scales (happy/sad, well/unwell, interested/bored, confident/not confident and optimistic/not optimistic) that was designed for use by those with mild-to-moderate levels of dementia. The CWS is conceptually based as a measure of wellbeing ‘in the moment’ rather than attempting to assess change over a long period of time. In the moment experiences are shorter-term experiences encountered by those living with dementia, family members and professional care staff trying to “fill as many of these moments with as much meaningfulness as possible” ( Killick, 2016). For the purposes of the present study, in the moment activities were all arts-based and occurred during a 60–120-minute time period.

Composite scores for the CWS range from 0 – 500 and subscale scores range from 0 – 100. A composite sum of each sub-scale is calculated (0 - 500) as an overall measure of in the moment subjective wellbeing. The CWS has been used in previous studies where it has demonstrated good internal consistency ( Camic *et al.*, 2019; Johnson *et al.*, 2017). Reliability analysis conducted on the composite scores of the CWS for the current data provided a score of Cohen’s *α* = 0.81, which shows good internal consistency.

 Because the respondent is not limited to predefined descriptors, VAS have been suggested to be more able to detect small levels of change ( Klimek *et al.*, 2017). VAS have also been found to be a valid and reliable measurement tool of subjective experiences ( Aitken, 1969; McCormack *et al.*, 1988). Furthermore, due to the ease of their construction and limited reliance on language and interpretation, VAS are quick to administer and score ( Klimek *et al.*, 2017; Little & McPhail, 1973; McCormack *et al.*, 1988), whilst being valid for use in repeated measures studies and able to detect change over a short period of time ( Wewers & Lowe, 1990). In addition, people with dementia have been shown to use VAS type scales in a similar way to the general population ( Arons *et al.*, 2013).

### Data analysis

Pre- and post-session composite and sub-scale scores of the CWS were calculated for all participants by subtracting the pre-session scores from the post-session scores. A positive score indicates an increase in wellbeing while a negative score indicates a decrease in wellbeing. Data was analysed using SPSS version 24. Initial analysis revealed requirements were not consistently met for parametric analysis and remained non-normally distributed when running corrections using logarithmic transformations (Kolmogorov-Smirnov >0.05) Therefore, non-parametric alternatives were used, including Wilcoxon-signed rank tests, Mann-Whitney U tests, Spearman’s correlations and Kruskall-Wallis tests. Pre- and post-session scores for the CWS composite and sub-scale scores were compared for a range of variables (carer present, intervention involved co-creativity, number of sessions attended). Results of G* power analysis for finding a small to moderate effect (Cohen’s *d* = 0.25) using Wilcoxon signed-rank test with α = 0.05 and power of 0.95 suggested a sample size of 183.

To test hypotheses 3 and 4 on whether the relationship between number of sessions and post-study wellbeing (composite CWS as well as subscales interested/bored, confident/not confident, optimistic/not optimistic, happy/sad and well/unwell) was moderated by co-creativity or carer presence, separate moderation analyses were completed. Baseline levels of the composite CWS as well as for each subscale were controlled for. Moderation analyses were conducted using model 1 of the PROCESS macro version 3.4 by Hayes (2019) with bootstrapping set to 5000 which has been found to be robust in cases of non-normality in data ( Hayes, 2009).

Although it was initially planned to analyse the data using structural equation modelling (SEM), the data were not normally distributed and SEM could therefore not be performed ( Shimizu & Kano, 2008).

## Results

### Wellbeing score changes (Hypothesis 1)

**Figure 1.**
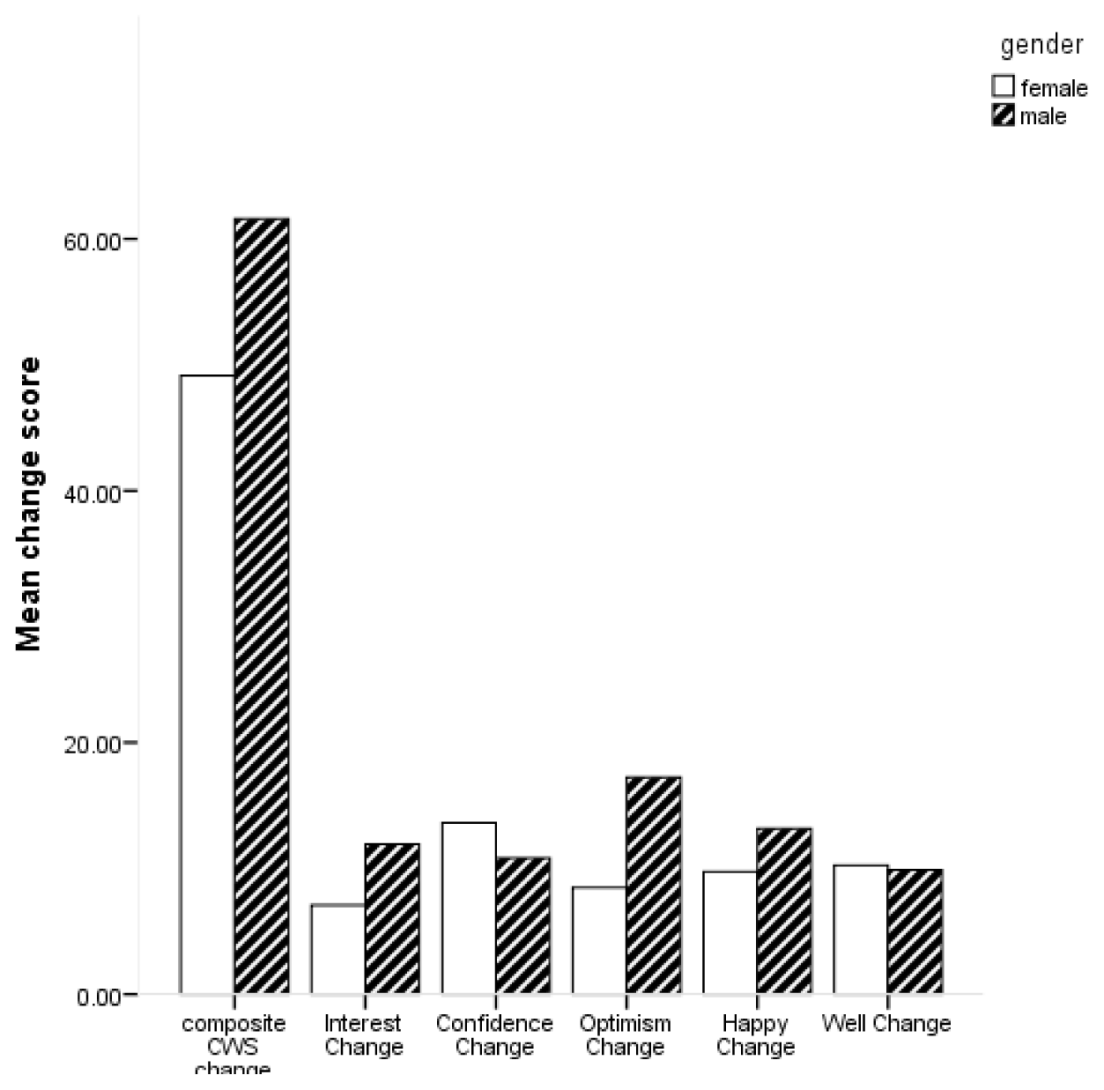
Bar chart of mean change scores on composite Canterbury Wellbeing Scales (CWS) and subscales by gender.

Descriptive statistics are shown in Table 3. Mean pre- post-session increases were observed for the composite and all subscale scores of wellbeing ( Camic *et al.*, 2021). The largest mean increase was observed for the optimistic/not optimistic subscale and the smallest mean increase was observed for the interested/bored subscale. The greatest increases in overall wellbeing were reported by male participants. Male participants reported the highest mean change in the optimistic/not optimistic subscale whilst female participants reported the highest mean change in the confident/not confident. Male participants reported the lowest mean change for the well/unwell subscale whilst for female participants, the lowest mean change scores were reported for the interested/bored subscale. See Figure 1 for mean scores on the composite CWS and subscales by gender.

**Table 3.** Descriptive statistics for the Canterbury Wellbeing Scales (CWS).

	Total (N=201)			Female (N=81)			Male (N=120)		
Scale	Mean	Range	SD	Mean	Range	SD	Mean	Range	SD
Composite change Pre-session Post-session	56.56 362.4 418.97	-70 – 200 173 – 500 220 - 500	52.66 75.7 66.65	49.14	-60 – 180	47.99	61.58	-70 – 200	55.22
Interest change Pre-session Post-session	9.97 77.23 87.15	-40 – 70 20 – 100 20 – 100	17.06 18.42 15.22	7.07	-40 – 40	15.59	11.92	-30 – 70	17.79
Confident change Pre-session Post-session	11.95 69.33 81.33	-60 – 70 10 – 100 20 – 100	18.16 21.34 16.66	13.63	-60 – 60	19.69	10.81	-43 – 70	17.05
Optimistic change Pre-session Post-session	13.7 68.43 82.03	-40 – 90 7 – 100 20 – 100	20.56 22.35 17.43	8.48	-40 – 80	20.47	17.23	-25 – 90	19.94
Happy change Pre-session Post-session	11.78 74.25 86.07	-30 – 60 10 – 100 25 – 100	15.87 18.49 15.81	9.73	-30 – 40	14.47	13.16	-30 – 60	16.66
Well change Pre-session Post-session	10.02 73.45 83.47	-70 – 77 7 – 100 30 – 100	18.32 19.34 16.55	10.23	-70 – 77	19.82	9.88	-50 – 60	17.32

The majority of participants reported increases in overall wellbeing as well as increases for all subscales of the CWS. The subscale with the largest number of participants reporting increases was for confident/not confident (N = 135) with the lowest observed for the well/unwell subscale (N = 119). Negative change (N = 14) and no change was also reported for overall wellbeing (N=15). The largest number of participants reporting negative change (N = 28) and no change (N = 57) was observed for the interested/bored and well/unwell subscales, respectively ( Table 4).

**Table 4.** Number of participants with positive, negative and no change scores for the Canterbury Wellbeing Scales (CWS).

	CWS					
Change	Composite	Interested	Confident	Optimistic	Happy	well
Positive	172	122	135	128	134	119
Negative	14	28	24	26	21	25
No change	15	51	42	47	46	57

Exploratory analysis was conducted to see if there was an association between gender and change scores by conducting a Mann-Whitney U test. A significant difference between female and male participants was found for the optimistic/not optimistic sub-scale of the CWS. Further exploratory analyses were conducted to explore any associations between age and change scores for wellbeing. To determine this, a Spearman correlation was performed. No significant associations between age and change scores for any subscales of the CWS were observed. Table 5 shows the results of the above tests.

**Table 5.** Wilcoxon-signed rank test for pre- post-session differences in subjective wellbeing; Kruskal-Wallis test for differences in subjective wellbeing for different types of ABI; Mann-Whitney U test of gender comparisons in pre- post-session differences in subjective wellbeing and Spearman’s correlation coefficients for association between age and change scores.

Test	Composite CWS	Interest	Confident	Optimism	Happy	Well
Mann-Whitney ( *U*)	4226	4144	4222.5	4722.5 **	4492	4479.5
Spearman ( *r _s_*)	-0.05	0.04	-0.05	-0.13	-0.004	0.07
Wilcoxon Signed-rank ( *T*)	16630 ***	9563.5 ***	11109.5 ***	10555.5 ***	10987 ***	8972.5 ***
Kruskal-Wallis ( *H*)	12.22	7.38	3.74	19.11 **	13.43	6.8

* *p* <0.05; ** *p*<0.01; *** *p* <0.001; ABI – Arts-based intervention, CWS – Canterbury Wellbeing Scales.

### Wellbeing results for different types of ABI

To test the hypothesis that wellbeing scores will increase following all forms of ABI, a Wilcoxon-signed rank test was conducted. Following all types of ABI, participants showed significant increases in the composite scores of the CWS with a large effect. Significant increases, with medium to large effect sizes, were also observed for the interested/bored, confident/not confident, optimistic/not optimistic, happy/sad and well/unwell sub-scales of the CWS. Consequently, hypothesis one can be accepted.

Further Kruskal-Wallis analysis was conducted to examine if there were differences between specific types of ABI and increases in wellbeing. The findings indicate that there was a significant difference in change scores for the optimistic/not optimistic subscale. Post hoc pairwise comparisons highlighted these significant differences to be between ABI involving object handling and art viewing against singing ( *M* = -19.43; SD = 4.5; *p* <0.001). Similarly, object handling and art viewing produce a more significant change in optimism than music/dance ( *M* = -23.93; SD = 5.55; *p* =0.001). In other words, participating in ABI involving object handling and art viewing was associated with a significantly greater increase in the optimism subscale.

### Relationship between number of attended ABI sessions and wellbeing (Hypothesis 2)

A Spearman correlation was performed to explore if the number of ABI sessions attended was associated with change in wellbeing and how this differed between male and female participants ( Table 6). Composite change scores did not significantly correlate with the number of sessions attended and neither did any of the subscales for the total sample. A significant small to moderate positive correlation was observed for female participants in the confident/not confident and well/unwell ( *r _s_* = 0.24, *p* <0.05) subscales.

**Table 6.** Spearman’s correlation coefficients for the association between change in wellbeing and the number of arts-based intervention sessions attended.

Scale	Correlation coefficient ( *r _s_*)
Composite Female Male	.07 .13 -.05
Interest Female Male	-.13 -.18 -.07
Confident Female Male	.13 .23 * .02
Optimistic Female Male	-.01 -.07 -.08
Happy Female Male	.03 .13 -.02
Well Female Male	.12 .26 * .02

* *p* <0.05.

The results provide limited evidence that the number of ABI sessions that participants attended relate to changes in wellbeing thus only partially confirming hypothesis 2. Moderation analyses were therefore completed to examine whether co-creativity in sessions or the presence of carers strengthened the relationship between number of sessions attended and wellbeing.

***Moderation analysis***

**Interaction between number of sessions attended and ABI involving co-creativity (Hypothesis 3)**

Descriptive statistics for change scores based on whether the ABI was co-created or not are shown in Table 7.

**Table 7.** Subjective wellbeing change scores for arts-based interventions that did/did not involve co-creativity.

	Co-created ( *N* = 75)			Not co-created ( *N* = 126)			Mann-Whitney U test		
Scale	Mean	Range	SD	Mean	Range	SD	*U*	*z*-score	*r*
Composite	54.99	-70 – 195	50.08	59.26	-60 - 200	54.33	4331.5	-0.84	.07
Interest	8.04	-30 – 50	18.18	11.21	-40 – 70	16.35	3964	-1.79	.09
Confident	12.61	-43 – 50	18.67	11.69	-60 – 70	17.94	4566	-0.25	.02
Optimistic	7.7	-40 – 47	16.41	17.13	-30 – 90	22.01	3483.5	-3.01	.22 **
Happy	11.61	-18 – 49	12.83	11.87	-30 – 60	17.51	4632.5	-0.75	.01
Well	12.58	-50 – 77	18.06	8.37	-70 – 60	18.37	3970	-1.78	.11

* *p* <0.05;** *p* <0.01.

**Figure 2.**
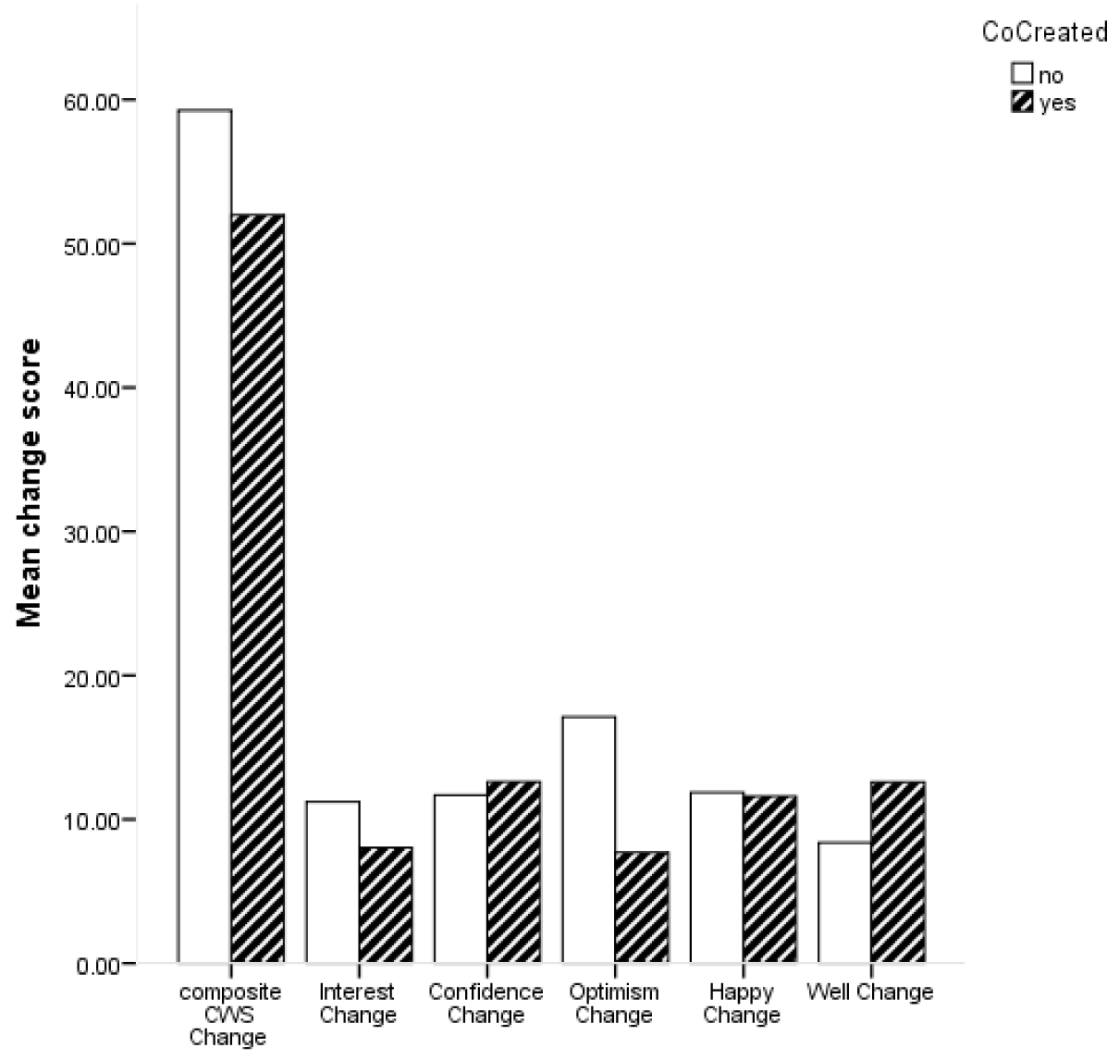
Bar chart for mean change in composite Canterbury Wellbeing Scales (CWS) and subscales for co-created and not co-created arts-based interventions.

Change scores for overall wellbeing were higher for ABI sessions that did not involve co-creativity compared to ABI sessions that were co-created. Change in the subscales interested/bored, optimistic/not optimistic and happy/not happy were consistent with this trend. Change in the subscales confident/not confident and well/unwell was larger in ABI that were co-created compared to ABI not co-created. Figure 2 shows the average change scores for the composite CWS and subscales for ABI which were co-created and those which were not.

Prior to moderation analysis, a Mann-Whitney U test was conducted (see Table 7). A total of 74 participants took part in an ABI that was considered co-created. Significant differences with small effect sizes were found for optimistic/not optimistic. No other significant differences were found for any other sub-scales or composite score.

Results of bootstrapped moderation analysis showed a significant interaction between number of sessions attended and co-creative ABI (the moderator). This means that the positive relationship between number of sessions attended and increased levels of overall wellbeing, as well as optimism, at post-ABI becomes stronger for those who experienced co-creation of their sessions (when controlling for respective baseline levels of wellbeing). Table 8 details interaction effects of moderation analysis. Hypothesis three can therefore only partially be confirmed. On the one hand, only one of the wellbeing subscales showed significant differences between co-created and non-co-created sessions and in a direction contrary to our expectations. However, the moderation analyses do confirm the positive role of co-creation on wellbeing through number of sessions attended. We believe the apparent contradiction between these results represented in Table 7 and Table 8 can possibly be reconciled with the consideration that co-creativity is a developing process that possibly takes time to establish, as this moderation analysis shows. Its positive effects are therefore significant only when participants experience co-creation for longer periods of time.

**Table 8.** Results of moderation with moderator co-creativity.

Number of sessions attended x co-creativity							
	F	∆R ^2^	*p*	*b*	SE _*b*_	t	95%C.I.
Interest	2.04	.01	.15	3.32	2.32	1.43	[-7.9, 1.26]
Confidence	1.59	.01	.21	3.04	2.4	1.26	[-7.78, 1.714]
**Optimism**	**6.56**	**.02**	**.01**	**6.77**	**2.48**	**2.73**	**[1.88, 11.65]**
Happiness	3.21	.01	.07	4.016	2.23	1.79	[-8.43, 0.4]
Well	2.71	.01	.1	4.08	2.48	1.65	[-8.97, 0.81]
**Composite CWS**	**4.65**	**.01**	**.03**	**16.84**	**7.81**	**2.16**	**[1.44, 32.33]**

*Note:* ∆R ^2^=adjusted R ^2^ change; *b=*effect size indirect effect; SE boot=bootstrapped Standard Error; 95% C.I.= 95% Confidence Intervals; significant results in bold. CWS – Canterbury Wellbeing Scales.

**Interaction between number of sessions attended and participants accompanied by a carer (Hypothesis 4)**

**Figure 3.**
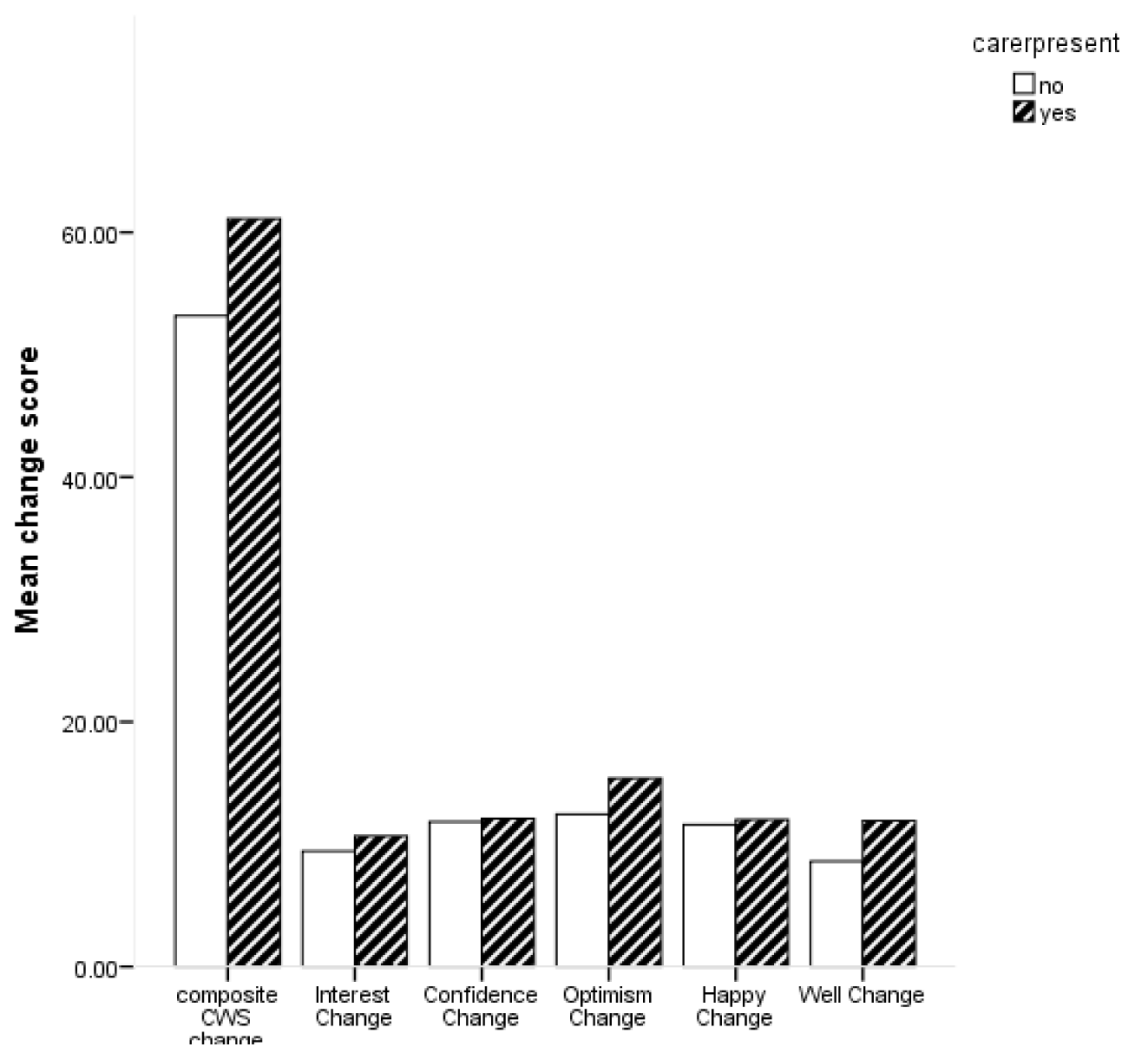
Bar chart for mean change in composite Canterbury Wellbeing Scales (CWS) and subscales for carer presence.

Descriptive statistics for change scores based on whether participants were accompanied by a carer or not are shown in Table 9. Overall change scores for wellbeing were higher for participants who were accompanied by a carer ( *M* = 72) compared to participants who were not accompanied by a carer. The same trend was observed for all subscales. A total of 86 participants were accompanied by a carer to the ABI. Participants who were accompanied by a carer also reported significantly higher levels of interest than participants who were not accompanied by a carer. Figure 3 shows the average change scores for the composite CWS and subscales for carer presence.

**Table 9.** Subjective wellbeing change scores for participants who were/were not accompanied by a carer.

	Carer present ( *N* = 86)			No carer present ( *N* = 115)			Mann-Whitney U test		
Scale	Mean	Range	SD	Mean	Range	SD	*U*	*z*-score	Effect ( *r)*
Composite	61.09	-70 – 200	57.04	53.18	-64 – 180	50.79	4640	-0.75	.08
Interest	10.66	-30 – 70	19.31	9.44	-40 – 50	15.24	4922	-0.06	.61 **
Confident	12.08	-50 – 70	19.21	11.84	-60 – 60	17.43	4912	-0.08	.01
Optimistic	15.38	-40 – 80	23.25	12.44	-30 – 90	18.29	4659.5	-0.71	.07
Happy	12	-30 – 50	15.72	11.61	-30 – 60	16.04	4834	-0.28	.01
Well	11.92	-70 – 77	20.8	8.6	-40 – 60	16.18	4380.5	-1.4	.09

** *p*<0.01.

Results of the bootstrapped moderation analysis showed no significant interaction between number of sessions attended and carer presence when controlling for baseline levels of wellbeing. Hypothesis four was therefore not confirmed. Table 10 details interaction effects of moderation analysis.

**Table 10.** Results of moderation between number of sessions attended and wellbeing with moderator carer presence.

Number of sessions attended x carer presence							
	F	∆R ^2^	*p*	*b*	SE _*b*_	t	95%C.I.
Interest	1.57	.01	.21	1.39	1.11	1.25	[-3.58, 0.8]
Confidence	1.08	.004	.3	1.17	1.13	1.04	[-3.4, 1.06]
Optimism	0.12	.001	.73	0.45	1.27	0.35	[-2.96, 2.07]
Happiness	.14	.001	.71	0.41	1.21.08	0.38	[-1.72, 2.53]
Well	.13	.001	.72	.42	1.19	0.36	[-2.77, 1.92]
Composite CWS	.003	<.001	.96	0.2	3.77	0.05	[-7.62, 7.23]

*Note:* ∆R ^2^=adjusted R ^2^ change; *b=*effect size indirect effect; SE boot=bootstrapped Standard Error; 95% C.I.= 95% Confidence Intervals.

### Relationship between specific dementia diagnosis and pre/post session wellbeing scores (Hypothesis 5)

 CWS change scores based on dementia diagnosis are displayed in Table 11. Participants diagnosed with frontotemporal dementia demonstrated the highest mean change in composite scores on the CWS.

**Table 11.** CWS change scores for dementia diagnosis.

Diagnosis		Composite	Interested	Confident	Optimistic	Happy	Well
Alzheimer’s disease (N=92)	Mean Range SD	62.27 -60 – 200 50.25	9.6 -40 – 50 15	11.89 -60 – 50 16.63	15.37 -25 – 90 22.83	14.57 -18 – 60 15.38	11.79 -10 – 77 15.55
FTD (N=17)	Mean Range SD	68.18 -30 – 180 55.76	13.13 -30 – 70 23.87	13.75 -20 – 70 20.04	14.06 -10 – 45 16.95	16.25 -10 – 40 14.55	17.81 -40 – 60 24.49
Vascular dementia (N=36)	Mean Range SD	52.54 0 – 160 44.99	9.11 -30 – 50 16.42	14.22 -10 – 50 16.88	9.27 -30 – 60 17.18	9.38 -20 – 50 14.9	10.19 -10 – 50 14.08
Mixed (N=35)	Mean Range SD	53.86 -64 – 195 64.15	12.17 -30 – 48 16.89	15.11 -43 – 60 18.69	15.17 -40 – 70 20.29	7.19 -30 – 49 17.33	5.05 -70 – 44 21.88
YOAD (N=5)	Mean Range SD	31.4 -70-110 65.66	7.8 -5-40 19.02	3.8 -5-20 10.71	11.2 -5-26 12.24	4.2 -5-12 7.19	4.4 -50-30 33.63
HIV (N=2)	Mean Range SD	42.5 25 - 60 62.58	2.54 0-5 3.54	5 0-107.07	12.54 10-153.54	104 0-2014.14	12.54 10-153.54
PCA	Mean	58.5	9	18	12.5	14	5
(N=2)	Range	10-107	-20-38	10-26	-10-35	8-20	0-10
	SD	68.59	41.01	11.31	31.82	8.49	7.07
PDD (N=1)	Mean Range SD	-30 - -	0 - -	-20 - -	0 - -	0 - -	-10 - -
SVD (N=1)	Mean Range SD	0 - -	-20 - -	-50 - -	-30 - -	-30 - -	-30 - -
LBD (N=1)	Mean Range SD	10 - -	10 - -	0 - -	0 - -	0 - -	0 - -

Key: FTD – Frontotemporal Dementia; HIV – Human Immunodeficiency Viruses; LBD - Lewy Body Dementia; PCA – posterior cortical atrophy; PDD – Parkinson’s Disease Dementia - SVD – Small vessel disease - YOAD – young onset Alzheimer’s disease.

To explore if there was an association between specific dementia diagnoses and pre- post-session changes to wellbeing, a between-group two-way ANOVA was performed including only participants for whom their specific diagnosis was recorded ( *N* = 189). There was no significant association between having a specific diagnosis and composite change scores of wellbeing (F(5, 183) = 0.65, *p* > .05). Similarly, there were no significant differences in change scores of either of the subscales. The results suggest that overall increases in wellbeing were not associated with a specific dementia diagnosis. Due to the small sample sizes for some diagnoses, specifically Parkinson’s related dementia ( *N* = 1), Lewy body dementia ( *N* = 1) and small vessel disease ( *N* = 1), it could not currently be confirmed if their wellbeing change scores are associated with specific diagnoses.

## Discussion

Using an existing database of attendance at various types of ABI, the current study aimed to explore which components of ABI are associated with wellbeing for people living with dementia. In summary, Hypothesis 1 that wellbeing scores increase following all forms of ABI was supported, Hypothesis 2 that the number of ABI sessions attended is positively associated with subjective wellbeing was not supported, Hypothesis 3 that the relationship between ABI and wellbeing scores at post-session was stronger for ABI involving co-creativity was ambiguous, Hypothesis 4 that the relationship between number of sessions attended and wellbeing scores at post-session is moderated by whether participants were accompanied by a carer was not supported and Hypothesis 5 that there is a significant relationship between the type of dementia and pre/post session changes in subjective wellbeing was not supported. The study did not seek to monitor long term effects of the interventions but was focused on more immediate ‘in the moment’ assessment ( Keady *et al.*, 2020). Considering the expressed need to find a range of alternative and/or complementary interventions to pharmacological management of dementias ( Camic *et al.*, 2018), the role of arts-based interventions is of increased interest. Previous research has suggested that ABI can have positive effects on wellbeing in addition to general health, cognitive processes and communication ( Ander *et al.*, 2013; Beard, 2021; Clare *et al.*, 2020; Young *et al.*, 2016; Zeilig *et al.*, 2014). Consistent with previous research, the overall results demonstrated that all forms of ABI had a positive association with wellbeing in this sample. The results also suggested that being accompanied by a carer could be an important factor during these activities and wellbeing was found to be associated with ABI that involve co-creativity. The current results do not sufficiently answer if increases in wellbeing are associated with the number of ABI sessions attended. Several methodological limitations mean that the findings should be interpreted cautiously, with further research warranted.

### Effectiveness of different types of ABI for wellbeing

Following all forms of ABI, a significant mean increase in wellbeing was observed with no significant differences observed between different types of ABI for the majority of wellbeing aspects, with the exception of optimism where a significant difference was found between ABI involving object handling and art viewing and singing. This is consistent with previous research into the influence of ABI on wellbeing of dementia participants (e.g. Camic *et al.*, 2019; Johnson *et al.*, 2017). These increases were observed for all subscales of the CWS as well as the composite score. The finding that these increases in wellbeing were generally not associated with gender or age suggests that there are potentially positive effects for anyone with early-to-middle stages of dementia. This is highlighted in the present study with 12.44% of participants being less than 65 years old. Dementia is generally seen as a syndrome affecting people of older age; however, more than 42,000 people in the UK are affected by young onset dementia, with symptoms starting before the age of 65 ( Rare Dementia Support, 2020). Possibly due to the larger number of older people with dementia, research has tended to focus on this demographic. However, it is possible that by doing so a significant number of people could be excluded from research, potentially leading to the assumption that ABI are not beneficial to them. Observations in the mean change scores for the subscales of the CWS also raises important implications in our understanding of wellbeing. The hedonic perspective of wellbeing emphasises the importance of positive affect ( Diener, 1984); however, in the current study the happy subscale did not demonstrate the largest mean change. This supports the notion that wellbeing is a multi-dimensional construct that encompasses both the hedonic and eudaimonic approaches ( Diener, 2009).

***Co-creative ABI.*** Findings of moderation analyses showed that co-creativity of ABI significantly strengthened the relationship between number of sessions attended and overall (composite) wellbeing as well as optimism. This finding corresponds with previous research where those participating in an ABI showed higher levels of optimism ( Bourne *et al.*, 2019). Additionally, although ABI involving co-creativity consistently demonstrated smaller increases in mean change scores, these differences were not significant. This could be explained by the differences in sample sizes for each condition, with a larger number of participants taking part in ABI that did not involve co-creativity. In addition, the processes involved in co-creativity may take time to develop over multiple sessions. Alternatively, it could be partly explained by the difficulties in defining co-creativity ( Zeilig *et al.*, 2019). Although it is suggested that the defining characteristics of co-creativity is a shared process, ownership and reciprocity, arguably all forms of ABI included in the current study involve some form of co-creativity. To explore the concept of co-creativity further and its influence on wellbeing, further research is necessary in order to avoid potential Type 2 errors.

***Carer presence.*** Next, no significant moderation effects were found between carer presence and number of sessions attended for wellbeing outcomes. This is at odds with previous findings in this area ( Bourne *et al.*, 2019; Isserow, 2008; MacPherson *et al.*, 2009; Zeilig *et al.*, 2019) that suggested being accompanied by a carer was a key component. Whilst the importance of social relationships and the supporting role of informal carers has been established as contributing to subjective wellbeing ( Lucas & Dyrenforth, 2006), it may be this is less of a factor in ABI over time. The interest, opportunities and engagement that different art activities offer may *possibly* offset being accompanied by a carer. One plausible explanation for this was offered by a carer who observed that involvement of people with dementia in group-based arts activities forms a new “triangular relationship […] between you, the facilitator and the object and the object is art. So here are two people exchanging conversation…one of them is learning and the other is helping the learning, not just here I am with a problem” ( Camic *et al.* 2016, p. 1037). We are not suggesting that ABI takes the place of an accompanying carer, but rather, participation in these type of activities for those with dementia can be beneficial with or without carer presence. This could be explored further in future research.

 The limited number of significant moderating effects observed supports the suggestion that wellbeing is a multi-dimensional construct. Furthermore, it highlights the difficulties and complexity in defining and measuring wellbeing as well as the interventions that can affect it in this population. The effectiveness of ABI for different dementia diagnoses are explored next.

### Specific dementia diagnoses and wellbeing

The term dementia refers to a collection of diseases that affect memory, cognition and behaviour and has a significant impact on an individual’s daily functioning (Alzheimer’s Society, 2019). Different dementias are associated with different difficulties, particularly in the early-to-middle stages of the respective disease. Consequently, when considering ABI, it is important to understand if individuals with different diagnoses experience it differently. Observations from the current study found no differences in participation levels, attendance or reported difficulties with the arts activities across different dementia diagnoses. Nor were significant differences in changes to wellbeing based on diagnosis found, suggesting that it has the potential to be beneficial for people with different types of dementia. However, this suggestion is made very tentatively due to considerable sample size differences across different dementia diagnoses.

### Contributions to wellbeing theory

Dodge *et al.*’s (2012) challenges and resources approach to wellbeing suggest that in order to achieve a state of relative wellbeing, people will utilise their psychological, physical and social resources to overcome their challenges. Disproportionate challenges following diagnosis and throughout the progress of the disease are faced by those living with dementia, making the attainment of wellbeing, arguably, difficult. It is therefore of utmost importance to support people to develop and utilise individual resources to promote their wellbeing. Kitwood & Bredlin (1992) suggest that through a person-centred approach, it is possible for someone with dementia to achieve a relative state of wellbeing. ABI interventions that promote creativity ( Camic *et al.*, 2019), active engagement and participation are person-centred approaches to care and therefore may be able to support people to achieve periods of time (e.g. ‘in the moment’) when they experience relative wellbeing. Likewise, measurement tools influenced by positive psychology ( Stoner *et al.*, 2019) and developed with the involvement of people with dementia and carers, as the CWS was, also contribute to more person-centred care research. The results of the current study suggest that ABI promotes and positively effects wellbeing and are a valuable initiative that can provide psychological, physical and social resources needed to achieve this.

 The present study also lends support to the applicability of Dodge *et al.*’s (2012) understanding of wellbeing as “the balance point between an individual’s resource pool and the challenges faced” (p. 230), for people with dementia. We propose that arts activities can act as resources to support some of the psychological, social and physical challenges experienced by people with dementia. The “balance point” or “see-saw dips” may change more frequently across the course of a week or even within a day due to a range of challenges faced by this population, but wellbeing can nonetheless be strengthened when one’s resources can help re-create an equilibrium. In thinking about “how people cope with change and how levels of wellbeing are affected” ( Headey & Wearing, 1992, p.6), a lack of stimulation and engagement for those with dementia can lead to challenges such as lowered interest, reduced confidence, lessened optimism, unhappiness and feeling unwell. Yet, the arts can offer real life tools for people with dementia to actively use “as decision makers, with choices, preferences, and the possibility of becoming masterful” ( Seligman, 2002, p. 3).

### Limitations

 The CWS comprises visual analogue scales, which have been suggested to be non-intrusive measures that can detect small levels of change over brief time periods ( Klimek *et al.*, 2017). Whilst intending to measure in-the-moment subjective wellbeing, it has been proposed that individual experiences influence how people respond to VAS ( Klimek *et al.*, 2017). Inevitably, due to the wide variation in characteristics, participants will have different experiences both recently and at different times in their life that will influence how they respond on the CWS. Finally, whilst it has been suggested that VAS have a limited reliance on language ( Little & McPhail, 1973), it is reasonable to suggest that individual differences in interpretation based on experience ( Klimek *et al.*, 2017) could have influenced how participants interpreted the scales ( Wewers & Lowe, 1990).

Only those within the mild-to-moderate range of impairment were included within this study and all were residing on their own or with a family member. The CWS would not be appropriate to use for those with more severe forms of impairment. In the present study, all participants appeared to understand the terms used in the CWS. Some requested clarification but no participant indicated they did not understand the terms. If MMSE scores were available for all, it would have been useful to examine these scores in relationship to cognitive impairment and the ability to successfully complete the CWS. For most participants dementia severity was classified using the global scores of the CDR or approximated from MMSE scores; unfortunately, scores for 35 participants were not available, making it imprudent to formally analyse these scores across the data set; this could be assessed in future research.

 Demographic information indicated that there were large differences in sample sizes based on gender, diagnosis, if participants were accompanied by a carer, if the session involved co-creativity and the number of sessions attended. Although the majority of participants had a diagnosis of Alzheimer’s disease, which is reflective of population prevalence, the findings may not be representative to other dementia diagnoses. The differences in the number of participants accompanied by a carer and in ABI involving co-creativity mean that results should be interpreted with caution and could explain the limited findings observed. Additionally, we did not report ethnicity or socioeconomic data because not all of our partner organisations agreed to collect this information from participants due to concerns about privacy and intrusiveness. This was unfortunate as it may have provided more insight into who takes up arts-based interventions and their impact on different groups of UK residents. There is also the limitation of possible selection bias, as in most cases individuals chose to come to ABI rather than through random assignment to arts or non-arts intervention. Finally, the lack of a control group in the current study also limits the inferences that can be made. For example, it cannot be suggested that ABI perform any better than other forms of interventions for this population.

 Although there are limitations to the current study and to the study of ABI in dementia more generally, research in this area is still relatively in its infancy. The current research adds to the existing literature and encourages its ongoing exploration. Additionally, while the sample in the current study may be described as moderate in size, it is still a larger sample than has been generally used in ABI-related studies and offers the opportunity to accumulate data from different interventions and locations.

### Implications for dementia care

 Based on the findings, it is suggested that ongoing ABI are useful community activities as part of dementia care and should be considered within the context of social prescribing ( Chatterjee *et al.*, 2018) and public health initiatives ( Fancourt & Finn, 2019). Stronger links between such arts-based community activities and dementia healthcare services could increase awareness of ABI and encourage clinicians to recommend them as interventions to support wellbeing for this population. The findings also support the assertion that people with dementia are important informants of their own experiences ( Kaufmann & Engel, 2016), where their subjective experiences are acknowledged as their reality ( Brooker, 2003). By valuing subjective experiences of those affected by a dementia there can be a greater shift towards person-centred care and helping to maintain personhood ( Kitwood, 1997). In addition, the CWS is an easily administered and scored questionnaire and may prove useful for care staff and family carers.

### Research implications

 The current study suggests that further research is warranted to better understand how ABI can affect wellbeing for those with dementia. In particular, in the moment experiences need to be better understood as situated “within a continuum of moments that could be used to contextualise and frame the lived experience of dementia” ( Keady *et al.*, 2020, p.1). How do arts activities relate, for example, to moments immediately preceding and after such activities? If “moments are centrally about a shift in perception” as Tipper (2013, p. 15) contends, how do arts activities shift perception within brief, one-to-two hour, in-the-moment experiences? How do people transition to the next moment? What happens in those following moments to wellbeing, satisfaction, behaviour, interest, confidence, among other variables? Contextualising in-the-moment ABI activities across time may help us to better understand the lived experience of those with dementia and their carers and how arts activities play a role. Future research could be expanded to incorporate focused ethnography ( Harding *et al.*, 2021) within a quasi-experimental, mixed methods design across different time periods to better appreciate the continuum of in-the-moment experiences (e.g. across a single day or across sections of days over weeks or months). Conceptualising the continuum of moments as Keady *et al.* (2020) propose as “creating the moment; being in the moment; ending the moment; and reliving the moment” (p.7) may help to better connect moments of arts activities to other moments across specified time periods and not see them as isolated events. Another point worth considering in future research is whether the different sizes of groups of ABIs could have had an effect on outcomes. For instance, participating in relatively individualised or small-group settings may have had a different effect on wellbeing measures than larger groups, such as singing in a choir. This may have influenced results in addition to or aside from the type of ABI participants took part in. Therefore, this is worth considering once further research on different sizes of ABI groups has been conducted.

## Conclusions

The current study is one of the largest undertaken to date involving arts-based interventions and supports previous research that make positive associations between subjective wellbeing and arts-based interventions. In particular, a significant finding indicated that co-creative ABI strengthen the effect between the number of sessions attended and wellbeing outcomes, in particular optimism. Further research is needed to explore how carer-person with dementia relationships could be strengthened by co-participatory experiences and how participating over time might impact wellbeing of the dyad. Nevertheless, it is recommended that community-based arts activities are promoted as public health resources, which could lead to improved understanding of how to stimulate health and wellbeing for this population. Future research should consider longitudinal designs, larger sample sizes, some form of randomisation where ethically feasible, and between-subjects analysis in order to better understand social and psychological mechanisms of these interventions.

## Data availability

### Underlying data

Zenodo: Canterbury Wellbeing Scales (CWS) raw dataset. https://doi.org/10.5281/zenodo.4468613 ( Camic *et al.*, 2021).

This project contains the following underlying data:

- Canterbury Wellbeing Scales raw dataset.xlsx (Raw data from arts-based programs that were analysed in the present study including composite Canterbury Wellbeing Scales (CWS) scores and scores from all five subscales. Also included are the types of venues the interventions took place and the corresponding activity (intervention), whether a carer was present, number of sessions and whether the sessions were co-created. Demographic information including type of dementia and years diagnosed, age and gender are also provided.)

### Extended data

Zenodo: Canterbury Wellbeing Scales: directions and scales. https://doi.org/10.5281/zenodo.4063768 ( Camic, 2020).

This project contains the following extended data:

- Canterbury Wellbeing Scales.pdf (Directions for use and scales)

Data are available under the terms of the Creative Commons Attribution 4.0 International license (CC-BY 4.0).

## Footnotes

^1^ For the purposes of this paper, the term “wellbeing” will be used throughout.
