# Supplementary material for: Arts-based interventions for people living with dementia: Measuring ‘in the moment’ wellbeing with the Canterbury Wellbeing Scales
Source: Wellcome Open Res. 2021 Sep 9;6:59. Originally published 2021 Mar 15. [Version 3] doi: 10.12688/wellcomeopenres.16596.3 (PMC8370129; doi:10.12688/wellcomeopenres.16596.3)
Supplement: Peer review history

# Peer review: Reviewer response for version 3

**DOI:** 10.21956/wellcomeopenres.19011.r45806

**Published:** 2021-09-10

**Authors:** Arthur Schall

**Affiliations:** 1 Institute of General Practice, Goethe University, Frankfurt am Main, Germany

I would like to thank the authors for the thorough response to my comments. The revised manuscript is an excellent addition to this field of research.

Is the work clearly and accurately presented and does it cite the current literature?

Yes

If applicable, is the statistical analysis and its interpretation appropriate?

Yes

Are all the source data underlying the results available to ensure full reproducibility?

Yes

Is the study design appropriate and is the work technically sound?

Yes

Are the conclusions drawn adequately supported by the results?

Yes

Are sufficient details of methods and analysis provided to allow replication by others?

Yes

Reviewer Expertise:

I'm musicologist, art historian and psychologist at the Goethe University Frankfurt (Geriatric Medicine, Psychogeriatrics & Clinical Gerontology, Institute of General Practice). My research interests include communication and quality of life in people with dementia, psychosocial treatment and training concepts as well as creative therapeutic interventions in dementia (esp. art and music therapy).

I confirm that I have read this submission and believe that I have an appropriate level of expertise to confirm that it is of an acceptable scientific standard.

# Peer review: Reviewer response for version 2

**DOI:** 10.21956/wellcomeopenres.18593.r44998

**Published:** 2021-08-16

**Authors:** Arthur Schall

**Affiliations:** 1 Institute of General Practice, Goethe University, Frankfurt am Main, Germany

This is a very detailed and well-written paper, and its contribution to research literature on the important topic of the measurement of well-being in people with dementia in the context of art-based interventions is valuable. The introduction is highly informative, and the pertinent literature is reviewed. The results are well structured with respect to all five hypotheses. The discussion section merges the results coherently, and relevant implications for dementia care and future research are drawn. There are only a very few minor issues that I would prefer to see addressed.

 Dementia severity was classified using CDR and MMSE scores (p. 8). The authors report mild-to-moderate impairment. It would be interesting to know the means and standard deviations for the included participants (as well as for the sub-samples), especially against the background of the enormous age range (31 - 95) and the many different dementia diagnoses.

 The language comprehension and verbal communication of people in the middle stages of dementia can be significantly impaired. I therefore wonder if all participants were able to understand and to differentiate between the terms used in the CWS subscales, e.g. happiness/ sadness vs. feeling well/unwell vs. being optimistic. Our research experience indicates that people with dementia with a MMSE score under 17/18 often have serious problems fully understanding such complex terms as for example “being confident”. Further details on MMSE scores (range) would be helpful in this context. Perhaps it would be possible to discuss these concerns.

 The study is based on a wide range of different art-based interventions and these are described in detail. Is it possible that there may have been differences in the effects on well-being of music-based interventions (singing) and art-based interventions in galleries and museums (e.g. due to various impact factors)? Or perhaps they existed between relatively individualised settings in very small groups (e.g. specialist science museum: 4 participants/multi-use creative space: 5 participants) and larger group settings such as singing in a choir (18 participants)? This point should be addressed in the discussion section.

 Finally, two formal aspects:

 Table 11 would benefit from an additional column providing the numbers of participants with the various types of dementia.

 p. 15: “… range of alternative interventions to pharmacological management of dementias”. I would prefer to speak of “complementary” interventions to pharmacological management.

Is the work clearly and accurately presented and does it cite the current literature?

Yes

If applicable, is the statistical analysis and its interpretation appropriate?

Yes

Are all the source data underlying the results available to ensure full reproducibility?

Yes

Is the study design appropriate and is the work technically sound?

Yes

Are the conclusions drawn adequately supported by the results?

Yes

Are sufficient details of methods and analysis provided to allow replication by others?

Yes

Reviewer Expertise:

I'm musicologist, art historian and psychologist at the Goethe University Frankfurt (Geriatric Medicine, Psychogeriatrics & Clinical Gerontology, Institute of General Practice). My research interests include communication and quality of life in people with dementia, psychosocial treatment and training concepts as well as creative therapeutic interventions in dementia (esp. art and music therapy).

I confirm that I have read this submission and believe that I have an appropriate level of expertise to confirm that it is of an acceptable scientific standard.

# Peer review: Reviewer response for version 2

**DOI:** 10.21956/wellcomeopenres.18593.r43804

**Published:** 2021-05-13

**Authors:** Tom Dening

**Affiliations:** 1 Mental Health & Clinical Neurosciences, School of Medicine, University of Nottingham, Nottingham, UK

I thank the authors for responding to my comments. I think they have dealt satisfactorily with all the points raised.

Is the work clearly and accurately presented and does it cite the current literature?

Yes

If applicable, is the statistical analysis and its interpretation appropriate?

Yes

Are all the source data underlying the results available to ensure full reproducibility?

Yes

Is the study design appropriate and is the work technically sound?

Yes

Are the conclusions drawn adequately supported by the results?

Yes

Are sufficient details of methods and analysis provided to allow replication by others?

Yes

Reviewer Expertise:

I am a clinical academic old age psychiatrist. My main research interests are in psychosocial aspects of dementia.

I confirm that I have read this submission and believe that I have an appropriate level of expertise to confirm that it is of an acceptable scientific standard.

# Peer review: Reviewer response for version 1

**DOI:** 10.21956/wellcomeopenres.18290.r43389

**Published:** 2021-04-14

**Authors:** Tom Dening

**Affiliations:** 1 Mental Health & Clinical Neurosciences, School of Medicine, University of Nottingham, Nottingham, UK

This is an interesting paper that makes a contribution to one of the challenges in arts and dementia research, which is how to capture the experience of participants in arts interventions close enough to the sessions to give a plausible estimate of the effects that the session has had for them. The paper investigates the use of an instrument called the Canterbury Wellbeing Scales (CWS) – the plural is used as there are five visual analogue scales that comprise the instrument.

 The paper proposes and tests five hypotheses about the effects of arts based interventions (ABI) on wellbeing scores, in this case the CWS. The dataset was drawn from nine completed studies that had used the CWS, and these involved a range of different arts based interventions, such as singing, dancing, museum object handling, and art making. There are a total of 201 complete CWS completed by people with dementia. The hypotheses tested included whether wellbeing scores improved across all forms of ABI, whether the number of sessions improved wellbeing, and whether the type of dementia was associated with the changes in wellbeing.

 The paper has an excellent introduction and a useful discussion of wellbeing, as well as sharing the concept of Keady *et al.* (2020) that ‘in the moment’ refers to a continuous set of moments moving forwards in time. This enables us to consider the experience within the session by inquiring about it as soon afterward as possible, which may be the best we can do unless we have the means of direct observation and recording within the session itself (e.g. Schneider *et al.*, 2019). ^1^ The interventions are also well described.

 The results and analysis are quite complicated and perhaps could be clearer. I would like to see the subheadings pointing directly to which of the five hypotheses is being tested in that section of text. In Table 2, there seems to be an arithmetical error in the rows as to whether the sessions were co-created, as this just adds up to 220 not 221 as it should do.

 The last two sentences in paragraph 2 of the results seem to contradict each other, or else the second sentence is simply incorrect in that 21 and 42 are of lesser magnitude than 28 and 57, respectively.

 The relationship between the analyses shown in Table 5 and the sections of corresponding text is baffling. Two of these four exploratory analyses (Mann-Whitney and Spearman) are described in paragraph 3 of the results, but they occupy the third and fourth rows of the table, below the Wilcoxon and Kruskall-Wallis analyses that are described in the text on the following two pages. This seems illogical and the table should be amended to reflect the order of the text.

 Paragraph 4 of the results ends with the statement that ABI involving object handling and art viewing was associated with greater increases in optimism. However, this seems to contradict the previous sentence which also includes singing and music/dance, so it is unjustified to omit these music-based interventions from the concluding sentence.

 On page 11, there is an analysis of the relationship between the number of sessions attended and ABI involving co-creativity. The second sentence states that change scores were higher for ABI that did NOT involve co-creativity. However, in the last paragraph, there appears to be a positive relationship between co-creativity and wellbeing when the number of sessions are taken into account. These two observations seem paradoxical. If they are both correct, then it requires some explanation. The discussion acknowledges that identifying which ABI are co-creative and which are not is rather arbitrary, and so perhaps this makes hypothesis 3 more or less untestable. I wonder whether the second sentence of the results section in the abstract or the second sentence of the conclusions section are still accurate. I am not persuaded that the case regarding co-creative ABI has been made. (Which is not to say that the act of co-creation is not potentially valuable, just that the data do not support it strongly.)

 I think this comes down to: hypothesis 1 – supported; hypothesis 2 – not supported, hypothesis 3 – equivocal; hypothesis 4 – not supported; hypothesis 5 – not supported. It would be helpful to have a clear unambiguous statement of this summary. Otherwise, the discussion makes a good summary of the limitations and the future implications of this work. The case for wider use of the CWS is well made in this paper.

Is the work clearly and accurately presented and does it cite the current literature?

Yes

If applicable, is the statistical analysis and its interpretation appropriate?

Yes

Are all the source data underlying the results available to ensure full reproducibility?

Yes

Is the study design appropriate and is the work technically sound?

Yes

Are the conclusions drawn adequately supported by the results?

Yes

Are sufficient details of methods and analysis provided to allow replication by others?

Yes

Reviewer Expertise:

I am a clinical academic old age psychiatrist. My main research interests are in psychosocial aspects of dementia.

I confirm that I have read this submission and believe that I have an appropriate level of expertise to confirm that it is of an acceptable scientific standard, however I have significant reservations, as outlined above.

## References

[1] : Facilitation of positive social interaction through visual art in dementia: a case study using video-analysis.*Ageing Soc*.2019;39(8) : 10.1017/S0144686X1800020X1731-175110.1017/S0144686X1800020X31285637PMC6614029

# Response

**Published:** 2021-04-22

**Authors:** Paul Camic

**Affiliations:** University College London, UK

**Responding to Reviewer 1 (Prof Tom Dening).**

 Thank you for your close and careful reading of our manuscript. Your comments are very much appreciated and we have attempted to address them follows:

  

1. I would like to see the subheadings pointing directly to which of the five hypotheses is being tested in that section of text. In Table 2, there seems to be an arithmetical error in the rows as to whether the sessions were co-created, as this just adds up to 220 not 221 as it should do ***. Response:*** *We have made this explicit in the subheadings and corrected our mathematical error re co-created sessions; the total of 221 is correct (n=75 co-created).*
2. The last two sentences in paragraph 2 of the results seem to contradict each other, or else the second sentence is simply incorrect in that 21 and 42 are of lesser magnitude than 28 and 57, respectively. ***Response:*** *We have now clarified this to:* *The largest number of participants reporting negative change (N = 28) and no change (N = 57) was observed for the interested/bored and well/unwell subscales, respectively (* *Table 4* *).*
3. The relationship between the analyses shown in Table 5 and the sections of corresponding text is baffling. Two of these four exploratory analyses (Mann-Whitney and Spearman) are described in paragraph 3 of the results, but they occupy the third and fourth rows of the table, below the Wilcoxon and Kruskall-Wallis analyses that are described in the text on the following two pages. This seems illogical and the table should be amended to reflect the order of the text. ***Response:*** *We agree and apologise for the initial order. This has been corrected to match the text.*
4. Paragraph 4 of the results ends with the statement that ABI involving object handling and art viewing was associated with greater increases in optimism. However, this seems to contradict the previous sentence which also includes singing and music/dance, so it is unjustified to omit these music-based interventions from the concluding sentence. *We can see that this was not presently clearly **.*** ***Response*** *: It has been revised as follows:* *Post hoc pairwise comparisons highlighted these significant differences to be between ABI involving object handling and art viewing against singing ( M = -19.43; SD = 4.5; p <0.001). Similarly, object handling and art viewing produce a more significant change in optimism than music/dance ( M = -23.93; SD = 5.55; p =0.001).*
5. On page 11, there is an analysis of the relationship between the number of sessions attended and ABI involving co-creativity. The second sentence states that change scores were higher for ABI that did NOT involve co-creativity. However, in the last paragraph, there appears to be a positive relationship between co-creativity and wellbeing when the number of sessions are taken into account. These two observations seem paradoxical. If they are both correct, then it requires some explanation. The discussion acknowledges that identifying which ABI are co-creative and which are not is rather arbitrary, and so perhaps this makes hypothesis 3 more or less untestable. I wonder whether the second sentence of the results section in the abstract or the second sentence of the conclusions section are still accurate. I am not persuaded that the case regarding co-creative ABI has been made. (Which is not to say that the act of co-creation is not potentially valuable, just that the data do not support it strongly.) ***Response***: We have re-worded the paragraph to more clearly convey our findings as follows: *Results of bootstrapped moderation analysis showed a significant interaction between number of sessions attended and co-creative ABI (the moderator). This means that the positive relationship between number of sessions attended and increased levels of overall wellbeing, as well as optimism, at post-ABI becomes stronger for those who experienced co-creation of their sessions (when controlling for respective baseline levels of wellbeing).* *Table 8* *details interaction effects of moderation analysis. Hypothesis three can therefore only partially be confirmed. On the one hand, only one of the wellbeing subscales showed significant differences between co-created and non-co-created sessions and in a direction contrary to our expectations. However, the moderation analyses do confirm the positive role of co-creation on wellbeing through number of sessions attended. We believe the apparent contradiction between these results represented in Tables 7 and 8 can possibly be reconciled with the consideration that co-creativity is a developing process that possibly takes time to establish, as this moderation analysis shows. Its positive effects are therefore significant only when participants experience co-creation for longer periods of time.*
6. I think this comes down to: hypothesis 1 – supported; hypothesis 2 – not supported, hypothesis 3 – equivocal; hypothesis 4 – not supported; hypothesis 5 – not supported. It would be helpful to have a clear unambiguous statement of this summary. Otherwise, the discussion makes a good summary of the limitations and the future implications of this work. The case for wider use of the CWS is well made in this paper. ***Response:*** *In the Discussion we have re-written the first paragraph for clarity. We have chosen to use the term “ambiguous” for H3, which we hope is now better explained in regards to the points addressed above:* *In summary, Hypothesis 1 that wellbeing scores increase following all forms of ABI was supported, Hypothesis 2 that the number of ABI sessions attended is positively associated with subjective wellbeing was not supported, Hypothesis 3 that the relationship between ABI and wellbeing scores at post-session was stronger for ABI involving co-creativity was ambiguous, Hypothesis 4 that the relationship between number of sessions attended and wellbeing scores at post-session is moderated by whether participants were accompanied by a carer was not supported and Hypothesis 5 that there is a significant relationship between the type of dementia and pre/post session changes in subjective wellbeing was not supported.*
7. ***Response:****In the Discussion section (Co-creative ABI subsection), we have also added the following sentence: In addition, the processes involved in co-creativity may take time to develop over multiple sessions.*
